# Pattern of Blood Stream Infections within Neonatal Intensive Care Unit, Suez Canal University Hospital, Ismailia, Egypt

**DOI:** 10.1155/2014/276873

**Published:** 2014-10-20

**Authors:** Rania Mohammed Kishk, Mohamed Fouad Mandour, Rasha Mohamed Farghaly, Ahmed Ibrahim, Nader Attia Nemr

**Affiliations:** ^1^Microbiology and Immunology Department, Faculty of Medicine, Suez Canal University, Ismailia, Egypt; ^2^Clinical Pathology Department, Faculty of Medicine, Suez Canal University, Ismailia, Egypt; ^3^Occupational Medicine Department, Faculty of Medicine, Suez Canal University, Ismailia, Egypt; ^4^Pediatric Department, Faculty of Medicine, Suez Canal University, Ismailia, Egypt; ^5^Endemic and Infectious Disease Department, Faculty of Medicine, Suez Canal University, Ismailia, Egypt

## Abstract

*Introduction.* Blood stream infection (BSI) is a common problem of newborn in neonatal intensive care units (NICUs). Monitoring neonatal infections is increasingly regarded as an important contributor to safe and high-quality healthcare. It results in high mortality rate and serious complications. So, our aim was to determine the incidence and the pattern of BSIs in the NICU of Suez Canal University Hospital, Egypt, and to determine its impact on hospitalization, mortality, and morbidity. *Methods.* This study was a prospective one in which all neonates admitted to the NICUs in Suez Canal University hospital between January, 2013 and June 2013 were enrolled. Blood stream infections were monitored prospectively. The health care associated infection rate, mortality rate, causative organism, and risk factors were studied. *Results.* A total of 317 neonates were admitted to the NICU with a mortality rate of 36.0%. During this study period, 115/317 (36.3%) developed clinical signs of sepsis and were confirmed as BSIs by blood culture in only 90 neonates with 97 isolates. The total mean length of stay was significantly longer among infected than noninfected neonates (34.5 ± 18.3 and 10.8 ± 9.9 days, resp., *P* value < 0.001). The overall mortality rates among infected and noninfected neonates were 38.9% and 34.8%, respectively, with a significant difference. *Klebsiella* spp. were the most common pathogen (27.8%) followed by *Pseudomonas* (21.6%) and *Staphylococcus aureus* (15.4%). *Conclusion.* The rate of BSIs in NICU at Suez Canal University Hospital was relatively high with high mortality rate (36.0%).

## 1. Introduction

Infection rates are standard indicators of quality and safety in all healthcare settings all over the world [1]. The challenge of infection control policies and procedures is always to decrease the frequency of healthcare-associated infections (HAI). Consequently determining infection rates through a surveillance program is central step in both identifying problems and evaluating the implementation of any program [2]. Healthcare-associated infections are associated with increased morbidity, mortality, and economic burden (either direct or indirect) [3]. More than 2,000,000 HAI in children and adults were reported in the United States, resulting in prolonged hospitalization with increased healthcare costs each year [4, 5].

Healthcare-associated infections in the neonatal intensive care units (NICUs) are affected by many factors as endemic microbial flora, clinical techniques, and antibiotic stewardship policies. Consequently, neonatal infections became more than a challenge for pediatricians [6].

Blood stream infections (BSIs) caused by commensal species play important roles in nosocomial infections in the NICU, which poses difficulties in determining true pathogens from contaminants [7]. It also served as the single most important type of infection because of their high frequency (59%) and potential life-threatening consequences [8].

Monitoring neonatal infections is increasingly regarded as an important contributor to safe and high-quality healthcare [9]. However, only few studies described BSIs in Egyptian NICUs. The objectives of this study were to determine the incidence and the pattern of BSIs in the NICU of Suez Canal University Hospital, Ismailia, Egypt, and to determine its impact on hospitalization, mortality, and morbidity among those critical age group patients.

## 2. Methods

### 2.1. Setting

The NICU at Suez Canal University Hospital is a referral unit that is supplied with 26 incubators. The unit receives newborns from the obstetric department, several hospitals, and healthcare centers in Suez Canal region. A trained infection control link nurse is available at the unit at all time with average 1 nurse for 2 neonates. A detailed written policies and procedures for hand hygiene are made clear in the unit. Disinfectants dispensers filled with alcohol 70% were provided at hand-wash stations; however, clean towels for hand-drying were not sufficiently available. Sterile and clean nonsterile gloves were used during neonatal care according to the followed guidelines. No routine surveillance cultures were taken and however sometimes used when an outbreak was suspected.

### 2.2. Study Design and Patient Population

This study was a prospective one in which all neonates admitted to the NICUs in Suez Canal University Hospital between January 2013 and June 2013 were enrolled in the study. During these 6 months, 317 neonates were admitted to the unit with no evidence of infection by clinical assessment at the time of admission. Blood stream infections were monitored prospectively by the attending pediatrician staff and infection control specialist.

### 2.3. Definitions

Subjects who had no infection and/or were not in the incubation period at presentation and who developed infection more than 72 hours after hospitalization were considered to have nosocomial infection. The diagnosis of nosocomial blood stream infection was made by clinical findings, laboratory findings, and hemoculture. Clinical findings including hypothermia, hyperthermia, apnea, bradycardia, circulatory disorder, lethargy, hypotonia, and feeding difficulty and laboratory findings including leukocytosis, leucopenia, thrombocytopenia, a ratio of immature/mature neutrophils >0.25, and a C-reactive protein (CRP) value of >1 mg/dL were considered to be significant.

### 2.4. Laboratory Methods

For each neonate, two blood samples per patient (1 mL each) were drawn under sterile conditions at least 30 min apart from each other. All blood cultures were processed by the Microbiology Laboratory, Faculty of Medicine, Suez Canal University. Blood culture bottles, especially for neonates, were incubated for ten days at 37°C and subcultured every other day to sheep blood agar, chocolate agar, MacConkey agar, and mannitol salt agar plates (oxoid) and incubated at 37°C for 24–48 hours. Isolates of bacteria were identified by conventional biochemical and serological methods according to the Clinical Laboratory Standard Institute (CLSI) criteria [10].

### 2.5. Susceptibility Testing

The antibiotic susceptibility for isolated pathogens was determined on Muller Hinton (oxoid) by Kirby-Bauer disk diffusion method and interpreted according to the National Committee for Clinical Laboratory Standards breakpoint values [11]. A BSI was defined as isolation of at least one positive peripheral-blood culture, whereas in cases of coagulase-negative staphylococci infections two positive blood cultures were required [12].

### 2.6. Statistical Analysis of Data

Statistical Package for Social Sciences (SPSS) for Windows 15 program was used for statistical analysis of the results obtained in the study. Demographic properties in the study were assessed by “descriptive” statistical analysis. “Unpaired” *t*-test was used for comparison of groups.

## 3. Results

During the 6-month period started at January 2013 till June 2013, a total of 317 neonates were admitted to the NICU in Suez Canal University; of these neonates 114 (36.0%) died (Table 1).

During this study period, 115 (36.3%) developed clinical signs of sepsis and confirmed by blood culture as blood stream infection in only 90 neonates (78.26%) with 97 isolates. The general characteristics of the infected neonates are shown in Table 2. The most common reason for hospitalization was preterm delivery in patients with nosocomial infection. The other reasons for hospitalization included meconium aspiration syndrome, transient tachypnea or asphyxia, congenital cardiac disease, and congenital metabolic diseases.

In 43% of the patients who had a positive culture, there was no problem in antenatal history, but 57% suffered from preeclampsia, eclampsia, hypothyroidism, and cardiac problems in antenatal period.

**Figure 1:**
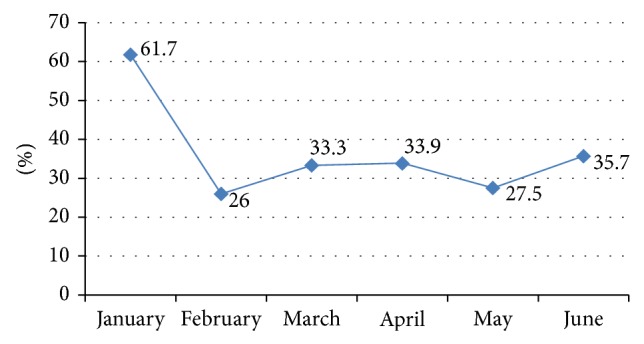
Frequency (%) of blood stream infections among admitted neonates during the study.

During this 6-month period of study, we noticed that the highest rate of death was in January although it has the lowest admission rate (29/47). The highest rate of admission was in March followed by April and June (57 versus 56 and 56 neonates, resp.) (Figure 1).

The most common clinical findings of blood stream infection were found to be apnea and bradycardia (45%), hypo/hyperthermia was found in 27%, circulatory disorder was found in 20%, and feeding intolerance was found in 17%. Laboratory findings included leukocytosis (64%), leucopenia (27%), thrombocytopenia (50%), an immature/mature neutrophils ratio of >0.25 (47%), and increased CRP (77%).

### 3.1. Microbial Causes of Blood Stream Infection

**Figure 2:**
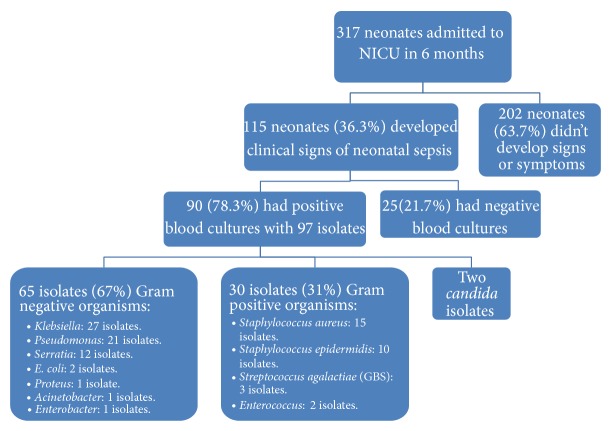
Study enrollment chart and pathogens recovered from neonatal blood culture.

The identified microorganisms in blood cultures are shown in Figure 2. A total of 65 isolates (67%) of 97 recovered isolates were Gram negative organisms, 30 isolates (31%) were Gram positive organisms, and only 2 isolates were* Candida*.* Klebsiella pneumoniae* were the most common pathogen accounting for 27.8% of the total isolates, followed by* Pseudomonas* (21.6%) and* Staphylococcus aureus* (15.4%). Although two bacterial sepsis episodes were observed more frequently in preterm babies, the difference was not statistically significant. Positive hemoculture was accompanied by pneumonia (35%), meningitidis (15.6%), or urinary infection (5%).

### 3.2. Complications of Infection

All infected neonates were on peripheral line cannulation or umbilical vein cannulation. Out of 90 infected neonates, the device related infection was significantly related to the length of the stay as 56.7% (51 neonates) were staying for more than 1 week in the incubator, 37.8% (34 neonates) for 2 days till up to 7 days, and only 5.5% (5 neonates) for 1 day.

### 3.3. Antibiotic Susceptibility

Aminoglycosides were found to be as efficient as third generation cephalosporins for* Klebsiella* species.* Pseudomonas* species were found to be highly sensitive to ciprofloxacin, imipenem, meropenem, and piperacillin-tazobactam and resistant to tobramycin, cefotaxime, and ceftriaxone as shown in Table 3.

*Serratia* species were resistant totobramycin, ciprofloxacin, imipenem, meropenem cefotaxime, and ceftriaxone. *Acinetobacter* spp. were found to be resistant to penicillin, aminoglycosides, and all cephalosporins.

*Staphylococcus aureus* was found to be sensitive to amikacin, vancomycin, teicoplanin, and linezolid with the highest degree and to oxacillin with the lowest degree. Coagulase negative staphylococci were found to be sensitive to vancomycin, linezolid, and teicoplanin. All the strains were resistant to ampicillin and penicillin (Table 3).

## 4. Discussion

Neonatal infections are an important cause of mortality and morbidity worldwide. In the World Health Organization 2000–2003 report, neonatal sepsis and pneumonia were responsible for about 1.6 million deaths each year, mainly in resource-poor countries [13].

In the present study, the total admitted neonates were 317; BSIs are considered one of the most important infections in our hospital (representing 28.4%) because of their potential life-threatening consequences that may end by death. Ho and his coworkers [8] considered BSIs the most common source of infection as they represented 59.0% and 5.6% of these were catheter-related [8]. On the other hand, in a recent study in Egypt, pneumonia was the most frequently occurring infection (11.3%) followed by blood stream infection (8.8%) in NICU [14] which comes in agreement with the two Brazilian studies [15, 16]. However, in developed countries the distribution of nosocomial infections in neonates is different, with BSI predominating, followed by pneumonia [15, 17, 18]. Most cases of BSIs in the NICU are associated with indwelling vascular catheters [19, 20]. Other risk factors may be associated with BSIs in the NICU as lipids administration, low birth weight, respiratory disease, catheter hub colonization, blood sampling for central venous catheters, and use of H2 blockers [21]. In another recent study for the prevalence of different nosocomial infections, Van der Zwet et al. studied one hundred and ninety-one neonates which developed 264 infections. They discovered blood stream infection is the most common nosocomial infection in NICUs (*N* = 138) followed by pneumonia (*N* = 69) [7].

Differences in BSIs rates between studies may be partially explained by the methodology and definitions used, especially in relation to the definitions of infection. The present study included patients only with microbiologically confirmed infections (90 neonates) although 115 neonates developed clinical signs of sepsis; this may partly explain why the infection rate appeared lower than in some other reports from developing countries, which included patients with clinical evidence of infection only [16].

In the present study, 115 neonates (36.3%) developed clinical signs of neonatal sepsis; 90 neonates (78.26%) of 115 neonates had positive blood cultures with 97 isolates. Van der Zwet et al. [7] and his team in 2005 mentioned that at least 25% of clinically diagnosed septic episodes are culture-negative [7]. This may be due to many factors as concurrent use of antibiotics and suboptimal sample volumes.

The incidence of neonatal sepsis is inversely proportional to birth weight, with incidences of sepsis in infants of birth weight <1000 g of 54.4%, compared with 33.3% in those weighing 1000–<1500 g and 12.3% in those weighting 1500–2500 gm and 0% in those neonates above 2500 gm. These results are compatible with Kaufman and Fairchild [22] who reported that 20% of very-low-birth-weight (<1500 g) preterm infants experience a serious systemic infection and even with advanced neonatal intensive care and antimicrobials, a threefold higher mortality was reported for these infants who develop sepsis than their counterparts without sepsis during their hospitalization [22].

In our study, Gram negative pathogens were responsible for most neonatal BSIs representing 67% (65 isolates out of 97 isolates).* Klebsiella pneumoniae* and* Pseudomona*s are the cause of nearly half of infections (73.8%) followed by* Serratia *(12 isolates),* E. coli* (2 isolates),* Proteus* (1 isolate),* Acinetobacter* (1 isolate), and* Enterobacter *(1 isolate). In another descriptive hospital-based study carried out for 12 months in the NICU of the Mansoura University Children's Hospital, the investigators reported that* Klebsiella* spp. were the most frequently isolated organisms followed by* E. coli* [14] which is consistent with the results of previous Egyptian studies [23, 24].

In 2002, Huang and his coworkers described *Acinetobacter baumannii* as nosocomial pathogens in adults, being also responsible for infections in neonates hospitalized in ICUs, causing pneumonia. It is a ubiquitous microorganism implicated in a number of outbreaks in ICUs [25]. One year later, in 2003, Villegas and Hartstein [26] mentioned that most of these outbreaks have been traced to environmental sources, such as mechanical ventilation equipment and air conditioners [26].

In comparison with previous studies, CoNS is the predominant source of infection in NICU in the United States and Asian countries [27, 28] which is quite different from the community-acquired setting. This is not compatible with our results as the most predominant Gram positive organism was* Staphylococcus aureus* representing 15% of isolated Gram positive organisms.* S. aureus* is the most common pathogen causing pustulosis and cellulitis in neonates. The presence of virulence factors such as the Panton-Valentine leukocidin is thought to contribute to the pathogen's ability to cause skin and soft tissue infections [29]. In addition to the underdeveloped epidermis and frequent breeches in skin integrity due to intravenous catheters, blood draws and heel sticks place preterm neonates at risk of infection [30]. With advances in our NICU, the survival rates, hospital stay, and number of invasive procedures have increased. This might be part of the reason why* S. aureus *became the dominant Gram positive pathogen during the last decade.

By contrast, in western countries, Gram positive bacteria have predominated in most studies with CoNS accounting for 57% of all nosocomial infections in Australia and Gram positive bacteria were the leading causes of nosocomial infections (70%) in North America, again with CoNS predominating (accounting for 48% of infections) [31]. In Europe, most of nosocomial infections (76.4%) were caused by Gram positive bacteria, with CoNS being the most frequently occurring pathogens [18]. Even in Brazil, CoNS were the most frequent nosocomial pathogens, accounting for 41.6% of isolates [15].

On the other hand, Ho et al. [8] concluded that BSIs and skin/soft tissue infections caused by commensal species play important roles in healthcare-associated infections in the NICU. An increased incidence of* S. aureus *and* A. baumannii *infection and a decreased number of CoNS infections were observed. In another recent study, of blood stream infections, 59% were caused by CoNS [7].

Out of 97 isolates, there were only 2 isolates diagnosed as candidal infection.* Candida *infections are a common cause of late-onset sepsis in the NICU and are associated with significant mortality and neurodevelopmental impairment [32]. One of the most important reasons in managing candidal infection in our NICU is the use of prophylactic fluconazole in very-low-birth-weight infants to prevent invasive candidiasis. The rationale for this strategy is to prevent fungal colonization in high-risk infants and reduce the invasiveness of the disease [33].

The difference in the species distribution in our study compared with others may partly be attributable to many factors as the gestational age and frequency of use of invasive devices. However, there are many structural factors that may also have affected the pattern of infections as ambient temperature and humidity conditions and the lack of automatic preparation of total parenteral nutrition.

We also found that the mean hospital stay is significantly longer in infected than noninfected neonates. This is consistent with many previously reviewed studies from developing as well as developed countries [16, 34–37]. As all infected neonates were found to 8 beon peripheral line or umbilical vein cannulation, we recommend strict measure for 9 elimination of this line cannulation which would produce an overall great improvement 10 in this high sepsis rate.

Priority should be given to periodic infection surveillance for NICUs, so that standardized infection rates can be used as a benchmark to drive improvements in the quality and safety of care. We recommend also local infection surveillance on NICUs in Ismailia regarding all nosocomial infections which is important to decrease the mortality rate and also to direct rational antimicrobial prescribing. Training programs on the standard precautions, personal protective equipment, and clinical samples collection and transport were implemented in our unit regularly. NICUs should also receive regular reports of antimicrobial susceptibility data from their infection control microbiology laboratory.

## 5. Research Directions

Research directions are as follows:

- to assess the awareness, approach, and practice of all healthcare workers in the NICUs. This will be followed by training programs for all the stuff members,
- to study the infection rate and device associated infection in Ismailia NICUs not only Suez Canal University Hospital as a multicenter study.

## Figures and Tables

**Table 1:** General characteristics of the admitted neonates (*n* = 317) during 6-month period.

Characteristics	Number (%)
Gender	
Male	194 (61.2%)
Female	123 (38.8%)
Birth weight	
<1000 gm.	104 (32.8%)
1000 gm–<1500 gm	143 (45.1%)
1500–2500 gm	48 (15.1%)
>2500 gm	22 (7%)
Discharge status	
Alive	203 (64.0%)
Died	114 (36.0%)

**Table 2:** Characteristics of the infected neonates (*n* = 90) within 6-month period.

Characteristics	Number (%)
Gender	
Male	59 (65.6%)
Female	31 (34.4%)
Birth weight	
<1000 gm.	49 (54.4%)
1000 gm–<1500 gm	30 (33.3%)
1500–2500 gm	11 (12.3%)
>2500 gm	0 (0)
Discharge status	
Alive	55 (61.1%)
Died	35 (38.9%)

**Table 3:** Antibiotic sensitivity of Gram negative organisms.

	*Klebsiella* (*n*: 27)	*Pseudomonas* (*n*: 21)	*Serratia* (*n*: 12)	*E. coli* (*n*: 2)	*Proteus* (*n*: 1)	*Acinetobacter* (*n*: 1)
	Resistance (*n*)	Resistance (*n*)	Resistance (*n*)	Resistance (*n*)	Resistance (*n*)	Resistance (*n*)
Beta-lactams						
Ampicillin	20	3	10	2	1	1
Piperacillin-tazobactam	18	1	12	1	0	1
Sulperazone	17	0	9	1	0	1
Cefotaxime	15	21	12	1	1	1
Ceftriaxone	19	18	12	2	1	1
Ceftazidime	18	3	12	1	1	1
Imipenem	1	1	11	1	1	0
Meropenem	1	1	10	1	1	0

Non-beta-lactam						
Amikacin	15	1	10	0	0	1
Gentamycin	3	20	8	2	1	1
Tobramycin	5	21	4	2	1	1
Ciprofloxacin	5	21	9	2	1	1
